# Insanity and Uterine Diseases

**Published:** 1881-07

**Authors:** 


### INSANITY AND UTERINE DISEASE.

A communication to the April 2 number of the *New York Medical Record*, claimed that the majority of cases of female insanity in any institution were probably due to untreated uterine disease. The suggestion was not entirely new, but the physciatry and gynæcology therein shown was entirely original. The assertion was supported by what purported to be statistics from the writer's experience in the New York City Lunatic Asylum. The patients with uterine disease either did not die or recovered from their uterine disease before death, and the pathologist of the same institution questions the statement of the previous writer in toto, and goes as far in denying the co-existence of uterine and mental disease as the former did in asserting it. Both these gentlmen are in error, and their mutual error is likely to lead to disastrous results, to neglect on the one hand of psychiatrical treatment, and on the other hand of gynæcological.—*Chicago Medical Review.*

